# Platelet phagocytosis in acute Dengue infection

**DOI:** 10.1111/bjh.70023

**Published:** 2025-07-21

**Authors:** Giuseppe Colucci, Nora Michel, Benoît Favre, Sènan Mickaël D'Almeida

**Affiliations:** ^1^ Outer Corelab Viollier AG Allschwil Switzerland; ^2^ Department of Hematology University of Basel Basel Switzerland; ^3^ Centre Médical du Lignon Genève Switzerland

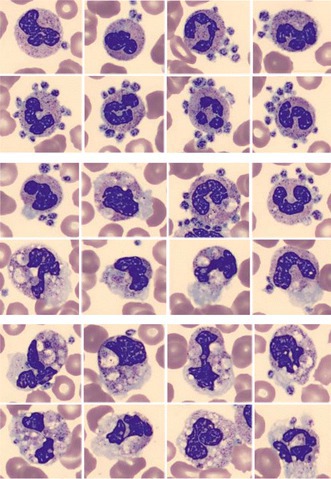

A 55‐year‐old woman, without significant medical history, presented with malaise, fever, myalgias and arthralgias 3 days after a trip to Martinique, in the eastern Caribbean Sea. Laboratory investigations showed bicytopenia (leucocytes: 1.9 × 10^9^/L; monocytes: 0.15 × 10^9^/L, lymphocytes: 0.32 × 10^9^/L, platelets: 121 × 10^9^/L). Haemoglobin was in the normal range (haemoglobin: 132 g/L).

Due to the Dengue outbreak in Martinique, a screening test detecting NS1 antigen was performed and resulted positive. Dengue antibodies Immunoglobulins M (IgM) and G (IgG) were negative. Molecular testing via polymerase chain reaction revealed a Dengue viral load of 10 million copies/mL, confirming a primary acute Dengue infection.

The peripheral blood film on the stand‐alone Digital Imaging System CellaVision® DC‐1 (CellaVision AB, Sweden) showed platelet satellitism around granulocytes (upper panel set, polymorphonuclear neutrophils with platelets on the surface, May–Grünwald–Giemsa stain, ×100 objective). At the same time, platelets merging with neutrophil granulocytes and attracting other granulocytes were observed (middle panel set, platelets merge with granulocytes, May–Grünwald–Giemsa stain, ×100 objective). Granulocytes with internalised and phagocytosed platelets showing multiple vacuoles are also seen (bottom panel set, granulocytes with platelets in the cytoplasm, May–Grünwald–Giemsa stain, ×100 objective). The patient received a supportive therapy and remained stable without other treatments. No organ dysfunction, bleeding nor haemorrhagic shock were observed. After 1 week, all symptoms resolved completely.

Platelet satellitism is a phenomenon in which platelets arrange themselves on the surface of other cells. It is generally observed in peripheral blood films of normal subjects prepared from blood samples anticoagulated with EDTA, in which platelets rosette around polymorphonuclear neutrophils. This rare phenomenon is frequently triggered in vitro by ethylenediaminetetra‐acetic acid (EDTA) in the presence of cryptic antibodies forming bridges between the glycoprotein IIb/IIIa complex of the platelet membrane and the neutrophil Fc gamma (FcgRIII) receptor. Commonly seen in EDTA, but not in samples treated with heparin or sodium citrate, platelet satellitism represents an in vitro cause of thrombocytopenia, that is, pseudothrombocytopenia, without clinical consequences. This phenomenon has also been observed in patients with vasculitis, lupus, mantle cell lymphoma, marginal zone B‐cell lymphoma and chronic liver disease.

In case of viral infection, thrombocytopenia can develop through several mechanisms. In Dengue infection, platelet satellitism represents the first step in platelets clearance through phagocytosis. It is also an in vivo and not only an in vitro phenomenon.

Dengue virus—a mosquito‐borne human viral pathogen—binds on the receptor dendritic cell‐specific intercellular adhesion molecule 3‐grabbing non‐integrin (DC‐SIGN), which is present on the primary target represented by dendritic cells and also by platelets.^1^ The binding between Dengue virus and platelets enhances phosphatidylserine expression by platelets, leading to apoptosis and to phagocytosis by macrophages.^2^

This remarkable case shows that the sequential binding of infected platelets around granulocytes, the internalisation and the phagocytosis process happen in vivo in the absence of antibodies.

## FUNDING INFORMATION

No funding has been required for this morphological case.

## ETHICS STATEMENT

No ethical approval was necessary for this case.

## PATIENT CONSENT STATEMENT

Written informed consent was obtained from the patient. The written consent is enclosed separately.

## PERMISSION TO REPRODUCE MATERIAL FROM OTHER SOURCES

No material is reproduced from other sources.

## CLINICAL TRIAL REGISTRATION (INCLUDING TRIAL NUMBER)

Not necessary, this is not a clinical trial.
